# The Use of the Buddy Balloon Technique in Transcatheter Aortic Valve Replacement Procedure

**DOI:** 10.7759/cureus.32568

**Published:** 2022-12-15

**Authors:** Muhammad Z Khan, Salil G Shah, Mohammed Murtaza

**Affiliations:** 1 Cardiology, Thomas Jefferson University Hospital, Philadelphia, USA; 2 Cardiothoracic Surgery, Thomas Jefferson University Hospital, Philadelphia, USA

**Keywords:** calcified aortic valve, severe aortic stenosis, aortic valve, buddy balloon, transcatheter aortic valve replacement

## Abstract

Transcatheter aortic valve replacement (TAVR) is a new, rapidly evolving technology used in severe cases of aortic stenosis. Some TAVR procedures have failed to deliver the prosthetic valve due to challenges. In these cases, it is difficult to pass a bulky prosthesis through a narrowed and calcified aortic valve. Special techniques may be needed in these challenging cases. In our case, the extreme calcification and the horizontal aorta caused the delivery system to hang up on the aortic valve. We used a buddy balloon from the contralateral femoral artery to assist in crossing the native aortic valve.

## Introduction

Transcatheter aortic valve replacement (TAVR) is frequently performed in patients at high or moderate surgical risk [1]. Patients with low surgical risk are also being investigated for TAVR. Although better catheters with smaller diameters and better valve designs have reduced procedure complexity, some situations remain challenging due to patient characteristics. In these cases, innovative methods may be required for safe treatments. A buddy balloon has been used in a few situations to help cross the aortic valve more easily. These situations have been mentioned in early TAVR and first-generation valve prosthesis experiences [2,3]. However, similar difficulties can be encountered despite improved versions of the valve prosthesis. We describe a situation in which a heavily calcified aortic valve and an angulated aorta make it challenging to implant the transcatheter valve. Our case differs from previous cases in that we used a low-profile peripheral balloon with a diameter of 7 mm rather than the bigger balloons.

## Case presentation

A 90-year-old male was referred for a TAVR operation because he had severe aortic stenosis and New York Heart Association (NYHA) class IV heart failure symptoms. His past medical history was significant for paroxysmal atrial fibrillation, critical aortic stenosis, permanent pacemaker implant, hypertension, hyperlipidemia, stage 3 chronic kidney disease, prostate cancer status post radiation therapy 17 years ago, an asbestos-related pleural disease requiring home oxygen (3-4 L), and anemia. The patient’s vital signs were within normal ranges, and a physical examination indicated bilaterally distributed 1+ pitting edema on the lower limbs with a grade 3/6 systolic ejection murmur at the apex. Transthoracic echocardiography revealed an aortic valve area of 0.63 cm^2^, and peak and mean pressure gradients of 88 mmHg and 53 mmHg, respectively. The Society of Thoracic Surgeons (STS) score for mortality was 19.5%, placing it in the high-risk category. The heart team evaluated the patient and deemed him to be inoperable. TAVR was recommended.

Procedure

**Figure 1:**
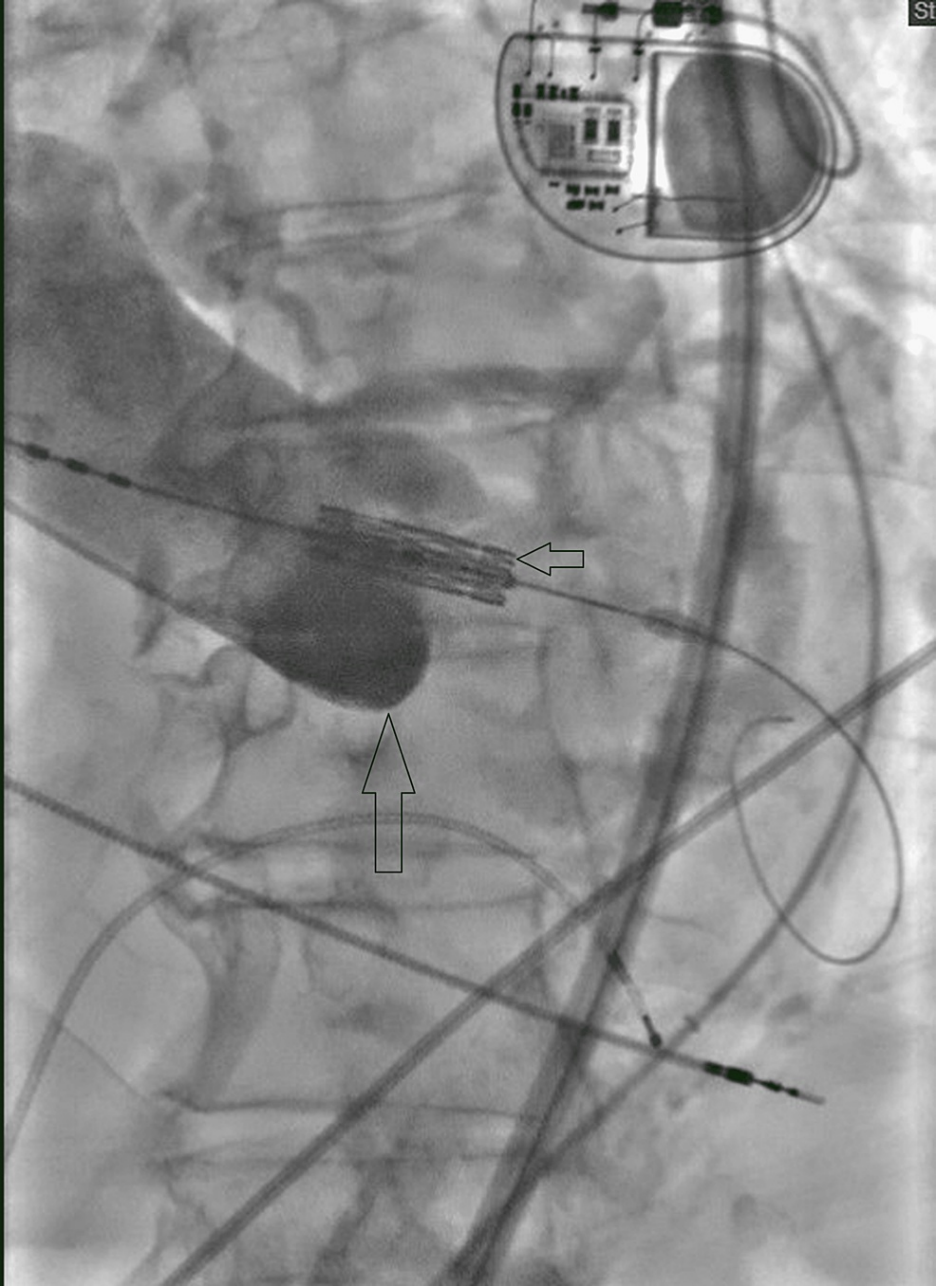
Deployment of the 26-mm Edwards Sapien Ultra valve The small arrow shows the Sapien valve, and the large arrow shows the aortic valve.

The procedure was performed under general anesthesia. Right groin access was used for valve deployment. Left groin access was used to place a temporary transvenous pacemaker. It was found that the patient’s iliac arteries were very calcified and tortuous. Placement of an Edwards sheath (Edwards Lifesciences, Irvine, CA, USA) was attempted; however, it was difficult to advance across an area of calcification near the aortoiliac bifurcation. A Lunderquist wire (Cook Medical, Bloomington, IN, USA) was placed. Pre-dilation with 14-French and then 16-French cook dilators facilitated the placement of Edwards sheath in the descending thoracic aorta. The initial aortogram was done with a 5-French pigtail catheter placed via the left femoral artery. An 18-mm Z-Med balloon (B. Braun Interventional Systems Inc., Bethlehem, PA, USA) was used for the pre-dilatation of the aortic valve. Then, a 26-mm Edwards Sapien Ultra valve (Edwards Lifesciences, Irvine, CA, USA) was advanced; however, we could not cross the aortic valve. Multiple attempts were made to change the catheter’s flexion, but this was unsuccessful. Low-pressure inflation of the aortic valve balloon failed to advance the valve. A JR4 catheter (Cook Medical, Bloomington, IN, USA) was introduced from the left groin after upgrading to 6-French sheaths. The JR4 catheter was used to place another Amplatzer extra stiff wire (Boston Scientific, Marlborough, MA, USA) into the left ventricle as a buddy wire, but this did not help either (Figure 1). The prosthetic aortic valve was positioned over the Amplatzer wire and pushed across the aortic valve while a 7 × 60 mm peripheral balloon was simultaneously inflated.

This technique was successful. The buddy balloon and Amplatzer wire were removed. The valve was deployed after pacing again at 180 beats per minute and confirming the position. The deployment balloon was pulled back into the descending thoracic aorta. An aortogram was then performed. The valve position was excellent, and no significant aortic regurgitation was observed. A transthoracic echocardiogram confirmed good valve position, gradient resolution, and minimal paravalvular aortic regurgitation. The echocardiogram performed the following day showed complete resolution of minimal aortic regurgitation. At the two-week follow-up, the patient is doing better, and his shortness of breath has improved.

## Discussion

The complexity of TAVR varies, and it is commonly used in the USA. Sometimes, innovative techniques are required to avoid failure. In our case, the prosthesis did not cross the aortic annulus because the iliac vessel was extremely convoluted, the aorta was horizontal, and the valve size was small. The buddy balloon approach was utilized by switching the angle of the valve and the delivery mechanism to make it easier to cross.

The TAVR technology is developing and poses numerous difficulties to experienced operators. Numerous issues, including interruptions and embolization of calcium ions to the brain and left ventricle perforation, might result from excessive pressing and pulling on the guide wire in the left ventricle [4]. According to studies, 2%-3% of TAVR surgeries are abandoned because the aortic valve is difficult to cross [4].

The native aortic valve can be difficult to cross for various reasons. Thick and calcified native leaflets, commissural fusion, and unfavorable approach angle caused by aortic tortuosity are some of the reasons that make it difficult to cross the native aortic valve [5]. Extensive commissural fusion of the right coronary cusp (RCC) and non-coronary cusp (NCC) appears to be a key factor in the increased difficulty of traversing the valve. Wires and catheters inserted via a transfemoral technique have the propensity to pass between the RCC and NCC and the outer line of the aortic arch and root [5,6]. The buddy balloon approach keeps the prosthesis away from the fused commissure by inserting an extra balloon into the commissure between the RCC and the NCC. Anatomical factors, including a dilated ascending aorta, a horizontal aorta, or a convoluted descending aorta, are other reasons for difficulty traversing the valve [4-7]. It seems that buddy wire may not be enough from the review of previous cases and our experience in this case. In our case, the significant calcium burden, the horizontal aorta, and the existence of substantial commissural fusion between the RCC and the NCC were challenges. The tortuosity of the iliac vessels made this worse.

Our scenario differs from other cases in that it uses low-profile environmental balloons instead of larger balloons [7]. In contrast to the 7 × 60 mm balloon employed in our research, 40 × 20 mm and 10 × 4 mm balloons were typically used as buddy balloons in earlier occurrences [7,8]. In our situation, this smaller balloon was sufficient to assist in crossing the valve. Therefore, this technique does not require larger balloons. In these high-risk patients, larger valvuloplasty balloons necessitate rapid pacing, which may increase procedure complexity.

## Conclusions

In conclusion, retrograde valve crossing and device advancement were difficult in our case, but the prosthesis could cross the valve with a smaller balloon. The buddy balloon technique could make the surgery simpler while also cutting down on radiation exposure, ensuring success, and reducing the possibility of complications.
